# CAR-T cell therapy in cancer immunotherapy – Biology, clinical successes, and emerging challenges: A review

**DOI:** 10.17305/bb.2026.14266

**Published:** 2026-05-26

**Authors:** Abdisa Tufa Bedada, Mohammed Mehdi, Ousman Mohammed

**Affiliations:** 1 Department of Biochemistry, College of Health Sciences, Addis Ababa University, Addis Ababa, Ethiopia; 2 Department of Medical Laboratory Sciences, College of Medicine and Health Sciences, Wollo University, Dessie, Ethiopia

**Keywords:** CAR-T cell therapy, cancer immunotherapy, chimeric antigen receptor, cytokine release syndrome, tumor microenvironment

## Abstract

Cancer immunotherapy has transformed oncology by enabling targeted activation of antitumor immune responses in patients with relapsed or refractory malignancies. Among adoptive cell transfer (ACT) strategies, chimeric antigen receptor T-cell (CAR-T) therapy has emerged as a pivotal therapeutic advancement, genetically redirecting T lymphocytes to recognize tumor-associated antigens independently of major histocompatibility complex (MHC) presentation. This review provides a comprehensive overview of the biological principles, design evolution, manufacturing platforms, clinical applications, resistance mechanisms, toxicities, and future directions of CAR-T cell therapy within cancer immunotherapy. Specifically, we examine the evolution of CAR architecture, spanning from first-generation constructs to advanced armored and fifth-generation platforms. Furthermore, we compare viral and non-viral gene delivery systems and discuss emerging approaches such as *in vivo* CAR engineering, allogeneic ”off-the-shelf” products, logic-gated receptors, safety switches, and alternative immune-cell platforms, including natural killer (NK) cells and macrophages. CAR-T cell therapy has achieved its most profound clinical success in hematological malignancies, particularly in cluster of differentiation 19 (CD19)-positive B-cell acute lymphoblastic leukemia and B-cell non-Hodgkin lymphoma, reporting durable remission rates of approximately 60–90% in specific clinical contexts. However, broader clinical translation, particularly in solid tumors, remains constrained by challenges such as cytokine release syndrome (CRS), immune effector cell-associated neurotoxicity syndrome (ICANS), antigen escape, tumor heterogeneity, poor trafficking, limited persistence, high manufacturing costs, and the immunosuppressive tumor microenvironment (TME). While next-generation strategies—including clustered regularly interspaced short palindromic repeats/CRISPR-associated protein 9 (CRISPR/Cas9)-mediated editing, lipid nanoparticle (LNP)-based messenger ribonucleic acid (mRNA) delivery, bispecific CARs, and inducible suicide switches—hold promise for improving safety, specificity, scalability, and accessibility, a significant number remain in preclinical or early-phase clinical development. Overall, CAR-T cell therapy represents a transformative “living drug” platform in oncology; however, its broader clinical utility is contingent upon improving durability, reducing toxicity, overcoming solid-tumor barriers, and validating next-generation technologies through robust, long-term clinical studies.

## Introduction

Cancer remains a leading cause of mortality globally, characterized by uncontrolled cell growth, local invasion, and metastasis [1]. Traditional treatment modalities, including surgery, radiotherapy, and chemotherapy, have been the cornerstone of cancer management for decades. While effective, these approaches are often non-specific, resulting in significant damage to healthy tissues and frequently failing to eradicate advanced or metastatic disease [2]. The recognition of the immune system’s pivotal role in identifying and eliminating malignant cells has paved the way for immunotherapy, a strategy designed to harness and enhance the body’s innate anti-tumor immunity [3]. Among the most promising advancements in immunotherapy is Adoptive Cell Transfer (ACT), which involves engineering and expanding immune cells *ex vivo* before reinfusing them into the patient [4].

Chimeric Antigen Receptor T-cell (CAR-T) therapy represents a revolutionary form of ACT that has generated remarkable responses in patients with otherwise untreatable malignancies [5]. This innovative therapeutic approach modifies T cells to express CARs that specifically target and destroy cancer cells. The U.S. Food and Drug Administration (FDA) has approved seven CAR-T cell products, each exhibiting varying effects and clinical outcomes. CAR-T therapies have garnered significant attention due to their ability to improve patient survival, achieving remission rates of approximately 80%–90% in specific hematological malignancies [7].

Since the initial conceptualization of CARs in the late 1980 s [8], the field has rapidly progressed. The first proof-of-concept in hematological cancers, particularly those targeting the CD19 antigen, culminated in the first FDA approvals in 2017 [9]. Despite the curative potential of CAR-T therapy for certain hematological malignancies, its clinical application remains largely confined to a narrow spectrum of targets, such as B-cell acute lymphoblastic leukemia and lymphoma. Most emerging CAR-T strategies targeting solid tumors remain in early-phase clinical trials or preclinical stages, highlighting a critical gap between technological innovation and clinical translation [6, 7]. Moreover, the application of CAR-T therapy to solid tumors has been hindered by challenges, including the immunosuppressive tumor microenvironment (TME), tumor antigen heterogeneity, and on-target/off-tumor toxicity [10, 11]. Furthermore, all available FDA-approved CAR-T cells are produced through the *ex vivo* modification of autologous T cells. This process involves isolating patient T cells, modifying them using lentiviruses or retroviruses, and reinfusing them into a lymphodepleted environment to enhance their survival, proliferation, and anti-cancer activity. Such methods are costly and complex, requiring individualized preparation, thus presenting significant barriers to the widespread adoption and accessibility of CAR-T therapy in clinical practice [6]. This review will examine the development, mechanisms, clinical applications, challenges, and future directions of CAR-T cell therapy, emphasizing its potential as a transformative “living drug” in oncology.

## The evolution and design of CAR-T cells

Synthetic biology has been instrumental in the development of chimeric antigen receptor architecture, which effectively combines the targeting precision of an antibody with the cytotoxic capabilities of a T cell. A canonical chimeric antigen receptor (CAR) comprises several key domains, each with distinct and essential functions. The extracellular portion presents a single-chain variable fragment (scFv) that imparts specificity for the targeted tumor-associated antigen, serving as a guidance system [12]. This extracellular domain is linked to variable hinge and spacer regions, projecting to the cell surface to facilitate interaction with the target antigen. The intracellular component mediates T-cell activation, while the transmembrane domain anchors the CAR within the T-cell membrane. The intracellular region is crucial for this process, with the CD3^ζ^ chain—derived from the native T-cell receptor complex—serving as the primary signaling component that initiates activation upon antigen recognition [13, 14].

The sophistication of CAR design has advanced through distinct generations, with each iteration enhancing potency, persistence, and safety [15]. The first CARs, developed in the late 1980s, contained only the CD3^ζ^ signaling domain [16]. Although capable of redirecting T-cell specificity, they exhibited limited anti-tumor efficacy and poor persistence, akin to T-cell anergy due to insufficient sustained activation and proliferation *in vivo*. Recognizing these limitations, second-generation CARs incorporated co-stimulatory signaling domains, such as CD28 or 4-1BB, alongside CD3^ζ^. This enhancement resulted in full T-cell activation, significantly improving proliferation, cytokine production, cytotoxicity, and long-term persistence *in vivo* [17]. Currently, all FDA-approved CAR-T therapies are based on second-generation designs, demonstrating their significant impact. The quest for increased potency has led to the development of third-generation CARs, which integrate two distinct co-stimulatory domains with CD3^ζ^, aiming to create a synergistic signaling effect [18, 19]. However, the clinical advantages of this design over its predecessor remain an area of ongoing investigation. Recent breakthroughs include the discovery of a powerful CAR enhancer that can be integrated into the genome, significantly boosting the activity and persistence of CAR T cells, with the potential to redefine future generations [18, 20].

The continuous evolution and refinement in CAR-T design have resulted in fourth-generation, or “armored,” CARs, signaling a shift from merely enhancing T-cell activation to actively remodeling the hostile TME [21, 22]. Fourth-generation CARs are engineered with additional transgenes for inducible or constitutive expression of immunomodulatory molecules, transforming T cells into advanced agents capable of universal cytokine-mediated tumor cell killing. Upon identifying target antigens, these CAR-T cells not only execute direct cytotoxicity but also function as *in vivo* micro-pharmacies, releasing a spectrum of cytokines such as IL-12, IL-18, or IL-15 [23, 24]. These cytokines bolster the innate immune system by recruiting and activating endogenous immune cells, such as macrophages and natural killer (NK) cells, fostering a pro-inflammatory state while countering local immunosuppressive factors like transforming growth factor beta (TGF-β). This dual mechanism addresses critical challenges posed by tumor antigen heterogeneity and immune escape, including the elimination of antigen-negative cancer cells [25–27].

Building on this foundation, advancements have led to fifth-generation CAR-T cells. These sophisticated constructs enable the activation of the Janus kinase/signal transducer and activator of transcription (JAK/STAT) pathway, in addition to CD3^ζ^ and co-stimulatory signaling, through the incorporation of full-length cytokine receptor domains, such as the IL-2 receptor β-chain, thereby further enhancing T-cell activation and anti-tumor efficacy [28]. This innovation effectively creates a “cytokine receptor knock-in,” providing potent, endogenous-like signaling that supports improved expansion and persistence of CAR-T cells. Additionally, these systems can be engineered to inducibly secrete pro-inflammatory cytokines, such as IL-12 and IL-18, upon antigen recognition, amplifying immune activation and enhancing anti-cancer effects [29]. CAR expression is typically achieved through viral vector-mediated transduction, resulting in semi-random genomic integration. However, emerging fifth-generation strategies utilize targeted genome editing approaches, such as clustered regularly interspaced short palindromic repeats (CRISPR)-mediated insertion into specific loci. A notable example is integration into the T-cell receptor alpha constant (TRAC) locus (T cell receptor alpha constant region), which disrupts endogenous T-cell receptor (TCR) expression and ensures uniform CAR expression while minimizing interference from native TCR signaling [29, 30].

Targeted integration into the TRAC locus enhances cellular stability, preserves T-cell identity, and improves functional persistence. This strategy also reduces the risks of T-cell exhaustion and graft-versus-host effects, thereby enhancing overall therapeutic efficacy and durability [30]. Furthermore, next-generation designs increasingly incorporate logic-gated systems and inducible suicide switches, allowing for precise control of CAR-T activity by necessitating the recognition of multiple tumor-associated antigens and enabling the controlled elimination of cells in cases of severe toxicity [31].

Several innovative strategies have emerged to address the limitations associated with autologous CAR-T cell therapy. For example, incorporating co-stimulatory domains (such as CD28 or CD137) into CAR-T cells can enhance their activation and functional responses following antigen recognition [32, 33]. Although cross-reactivity remains a challenge in CAR-T therapy, recent research has focused on developing CAR-T cells with improved specificity for antigens [34, 35]. These advancements involve designing restricted antigen-binding domains and selecting tumor-associated antigens with limited expression in normal tissues, thereby enhancing therapeutic efficacy, reducing off-target toxicity, and minimizing adverse effects. Optimized CAR designs can also improve T-cell proliferation and survival, limit activation-induced inhibitory signaling, and promote the development of memory T-cell phenotypes [7, 36].

Another promising approach involves utilizing allogeneic CAR-engineered cell products from healthy donors, which offer several advantages over autologous CAR-T cell therapy. These include the immediate availability of cryopreserved products, standardized production, increased time for cell modification, cost reduction, and improved scalability in the manufacturing process [37, 38]. However, the use of allogeneic CAR-T cells carries the risk of life-threatening graft-versus-host disease (GvHD). Innovative strategies have been initiated to mitigate these risks, which often include prolonged cytopenias, neutropenias, and frequent infectious complications associated with allogeneic CAR-T cell administration [39, 40]. Consequently, there remains a need for safer, more efficient, and effective engineered CAR-T cells that can eliminate cancer cells while simplifying production processes [41].

### *In vivo* CAR engineering for cancer immunotherapy

Recent advancements in *in vivo* CAR-T cell engineering present a promising solution to the challenges associated with both autologous and allogeneic *ex vivo* production [42, 43]. This approach facilitates the generation of immune cells within the patient using gene delivery systems, thereby eliminating the need for individualized manufacturing and streamlining the production workflow. This results in faster treatment delivery, reduced costs, and improved scalability while maintaining therapeutic efficacy. Furthermore, *in vivo* methods employing non-integrating vectors may minimize risks associated with permanent genetic changes, avoid T-cell depletion, and address safety concerns [44, 45].

Several strategies—including lipid nanoparticles (LNPs), polymeric nanoparticles carrying nucleic acids, virus-based delivery systems, and hybrid approaches—have been explored in preclinical models to enable *in vivo* generation of CAR-T cells. Each method presents distinct strengths and limitations, particularly concerning cell-specific targeting, potential systemic effects, and the risk of insertional mutagenesis. Among these, lipid nanoparticle–formulated mRNA delivery has emerged as particularly promising, bolstered by recent clinical successes in vaccine development and its potential for translating CAR expression into clinical practice [46]. Preclinical studies indicate that lipid nanoparticle–mRNA systems represent a viable alternative for cancer immunotherapy. The transient nature of CAR-T cells manufactured in this manner is advantageous, as it limits toxicity, allows for precise dosing, and does not necessitate lymphodepletion of the patient prior to administration. Most current strategies for *in vivo* CAR-T cell generation using LNPs involve incorporating antibodies or scFvs that target CD3, CD4, CD5, CD7, and CD8 into the lipid nanoparticles [45, 47, 48]. One study demonstrated that targeted LNP-mediated extrahepatic transfection achieved dose-dependent CAR expression in splenic T cells, induced effective cytokine release, and resulted in up to 90% depletion of B cells [46].

Viral-based delivery systems, particularly lentiviral vectors, are efficient for facilitating CAR expression through genomic integration, enabling long-term expression that is desirable for durable therapeutic applications. However, concerns regarding long-term safety and insertional mutagenesis remain significant. Although enhancements in pseudotyping and scFv targeting have improved T-cell selectivity, logistical complexity, immunogenicity, and regulatory challenges continue to limit widespread *in vivo* application [49].

**Figure 1.**
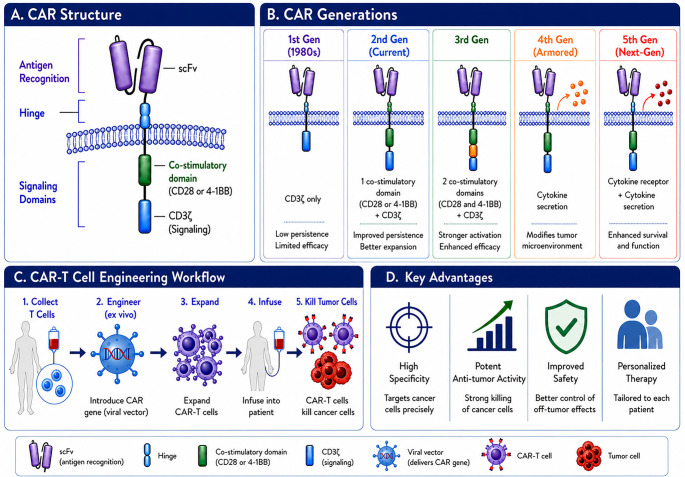
**Extending beyond T cells*: In vivo* engineering of immune cell therapies.** (A) Overview of basic CAR architecture, illustrating scFv-mediated antigen recognition alongside intracellular CD3^ζ^ and costimulatory signaling domains. (B) Progression of CAR generations, from first-generation constructs to FDA-approved second-generation CAR-T cells, culminating in advanced armored or fifth-generation platforms. (C) Patient T cells are collected, genetically engineered, and expanded *ex vivo* before being reinfused to target and eliminate cancer cells. (D) These strategies collectively enhance safety, potency, specificity, and personalized anti-tumor efficacy against cancer cells. Abbreviations: CAR, chimeric antigen receptor; CAR-T, chimeric antigen receptor T-cell; CD3^ζ^, cluster of differentiation 3 zeta; scFv, single-chain variable fragment.

Advanced approaches have been developed to create a more controlled environment for CAR-T cell generation by integrating immune cells, vectors, and stimulatory signals. For instance, one study utilizing CD8-targeted lentiviral vectors generated CD19-targeting CD8+ CAR-T cells *in vivo*, with 30%–50% of CD8+ T cells expressing the CAR and effectively eliminating Raji lymphoma cells in humanized NOD scid gamma (NSG) mice. However, therapeutic efficiency was compromised by myeloid phagocytosis and elevated cytokine levels associated with cytokine release syndrome (CRS) [50]. Similarly, the intraperitoneal administration of CD4-specific designed ankyrin repeat protein (DARPin) vectors transduced 40%–60% of CD4+ T cells within seven days. These cells exhibited greater proliferation and improved clearance of CD19+ B cells compared to CD8+ CAR-T cells, likely due to reduced exhaustion [49]. Despite these advancements, achieving spatial control along with efficient cellular activation and expansion remains challenging. Additionally, complexity, scalability, and clinical translation continue to hinder broader application of this technology [51, 52]. Off-target effects caused by viral and viral-derived delivery systems, including unintended signal transduction in non-target cells, also persist as significant challenges in *in vivo* gene engineering.

To address these challenges, emerging genome engineering platforms, such as Cas9-packaging enveloped delivery vehicles (Cas9-EDVs), enable precise gene editing with minimized off-target effects through predictive antigen-antibody interactions. This approach utilizes retrovirus-like particle-based Cas9-EDVs coupled with scFvs targeting T cells specific for CD3, CD4, or CD28. This method also facilitates the creation of allogeneic engineered T cells and mitigates the risk of GvHD by utilizing *in vivo* engineered CD19 CAR expression and knocking out the T-cell receptor alpha (TCRα) constant region [46]. Overall, the *in vivo* CAR-T cell engineering platform offers advantages in efficiency, specificity, and durability. However, none of these methods fully resolve the balance between safety, efficiency, specificity, and durability. Currently, lipid nanoparticle-based mRNA delivery provides superior safety, targeting, and sustained therapeutic effects [49].

Beyond T cells, analogous *in vivo* engineering principles apply to other immune cell populations such as NK cells and monocytes [53]. Among these, macrophages are particularly noteworthy due to their phagocytic nature, which enhances the internalization of nanoparticles and their effectiveness in *in vivo* transduction. Additionally, macrophages possess a natural ability to infiltrate cancer cells, with tumor-associated macrophages constituting a predominant cell type within the tumor microenvironment [54, 55]. Their active involvement in the tumor microenvironment enhances therapeutic effectiveness. Many studies have focused on synthesizing nanoparticles to reprogram macrophages, exploiting their tumor-homing properties. Consequently, *in vivo* CAR-macrophage therapies hold significant potential to enhance the antitumor activity of targeted cells by shifting their pro-tumorigenic M2 polarization to an anti-tumorigenic M1 polarization, facilitated by the release of pro-inflammatory cytokines and chemokines [25, 56]. Recently, CAR macrophages (CAR-Ms) have emerged as a promising immunotherapy for solid tumors, primarily due to their intrinsic tumor infiltration and effector functions. However, CAR-Ms are typically engineered using viral transduction, which is associated with potential immunogenicity and toxicity. To address these challenges, researchers have developed a bioinspired oxidized LNP platform for mRNA-based engineering of human CAR-Ms. Despite their preclinical advantages, these platforms remain largely investigational, with limited comparative clinical data against CAR-T therapies (Figure 1) [25, 57].

## Manufacturing and delivery of CAR-T cells

**Figure 2.**
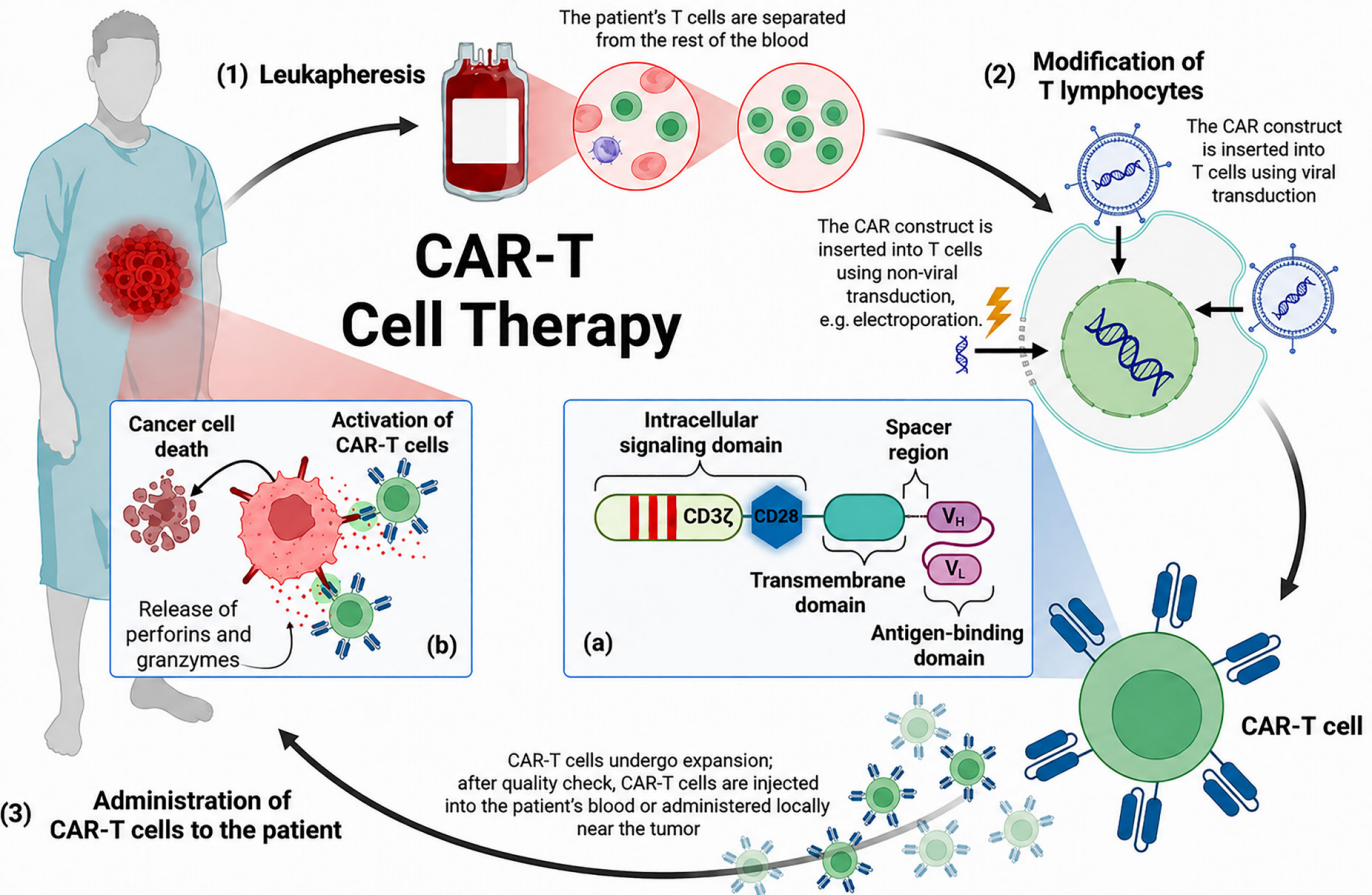
**Overview of CAR-T cell manufacturing and mechanism of action.** The figure summarizes the main steps of CAR-T cell therapy, including leukapheresis, *ex vivo* modification of patient-derived T lymphocytes using viral or non-viral gene delivery, CAR-T cell expansion, quality assessment, and reinfusion into the patient. The inset shows the modular CAR structure, composed of an scFv antigen-binding domain, spacer region, transmembrane domain, and intracellular CD3^ζ^/costimulatory signaling domains, such as CD28. After antigen recognition, CAR-T cells become activated and induce tumor-cell killing through perforin- and granzyme-mediated cytotoxicity, Fas/FasL signaling, and cytokine release. Adapted from Kowalczyk et al., 2024 [63]. Abbreviations: CAR, chimeric antigen receptor; CAR-T, chimeric antigen receptor T-cell; CD3^ζ^, cluster of differentiation 3 zeta; Fas/FasL, Fas/Fas ligand; scFv, single-chain variable fragment.

The production of CAR-T cells is a complex, multi-step process that integrates cellular immunology, genetic engineering, and clinical protocols. The process begins with leukapheresis, during which peripheral blood mononuclear cells, including T lymphocytes, are collected from the patient [58]. The collected T cells are subsequently activated using anti-CD3/CD28 stimulation to enhance proliferation and facilitate gene transfer. Various delivery systems, including viral vectors such as lentiviral or γ-retroviral systems, as well as non-viral approaches like transposon-based systems and mRNA electroporation, are under investigation as alternative strategies; however, only some methods enable stable genetic integration, while others result in transient expression (Figure 2) [59].

During CAR-T cell manufacturing, good manufacturing practice (GMP) conditions must be maintained to ensure the safety, identity, potency, and sterility of the product prior to infusion [60]. The therapeutic efficacy and persistence of CAR-T cells can be influenced by the composition of the cellular product, including the balance between CD4^+^ and CD8^+^ T cells and the proportion of memory subsets. Prior to infusion, patients typically receive lymphodepleting chemotherapy, commonly based on cyclophosphamide and fludarabine [61]. This regimen enhances CAR-T cell activity by eliminating endogenous lymphocytes, increasing the availability of homeostatic cytokines such as IL-7 and IL-15, and reducing immunosuppressive cell populations, thereby promoting CAR-T cell expansion and persistence *in vivo*. Finally, the engineered CAR-T cells are infused back into the patient, where they recognize and eliminate tumor cells expressing the target antigen [62, 63].

### Viral vector transduction

The choice of vector for gene delivery is crucial in CAR-T cell therapy, significantly influencing the characteristics of the final CAR-T cell product [64–66]. Gamma-retroviral and lentiviral vectors are the predominant platforms due to their capacity for stable genomic integration and sustained CAR expression; however, both systems do not fully mitigate the risks associated with insertional mutagenesis and manufacturing complexity [66, 67]. Mechanistically, gamma-retroviruses necessitate nuclear envelope breakdown during mitosis for genome integration, which restricts their activity to dividing cells. In contrast, lentiviral vectors utilize active nuclear import, allowing transduction of both dividing and non-dividing T cells [66–68]. While this capability provides lentiviral vectors with a functional advantage in preserving T-cell phenotype and flexibility, it does not eliminate safety concerns related to genomic integration. Furthermore, their integration patterns differ, with gamma-retroviruses favoring insertion near transcriptional start sites and regulatory regions, while lentiviruses integrate more frequently within transcription units, contributing to a relatively improved safety profile [66–69]. Despite the robustness and durability of viral systems in current clinical practice, their broader clinical implementation remains constrained by the aforementioned limitations [68–71]. Those limitations underscore the need for a non-viral delivery method that prioritizes safety, minimizes the risk of insertional mutagenesis, is cost-effective, easy to manufacture, has a high cargo capacity, and offers a prolonged production period.

### Non-viral delivery methods

In light of the limitations associated with viral delivery, non-viral vectors have emerged as a promising alternative for CAR-T cell engineering, offering enhanced safety profiles and reduced manufacturing complexity [72, 73]. Transposon systems, such as Sleeping Beauty (SB) and piggyBac (PB), present a more economical platform for stable gene integration via a “cut-and-paste” mechanism, although they also entail random integration [74]. CRISPR/Cas9 genome editing facilitates a more precise approach, enabling site-directed integration of the CAR transgene into specific genomic “safe harbor” loci, such as the TRAC locus [75, 76]. This method has been shown to produce a more uniform CAR-T cell product, enhancing both uniformity and potency. Additionally, electroporation of *in vitro* transcribed mRNA serves as a viable option for scenarios requiring temporary activity, such as targeting antigens expressed in healthy tissues to mitigate the risk of prolonged on-target/off-tumor damage [77, 78].

Overall, while viral vectors remain the most effective and clinically validated systems for CAR-T gene delivery, achieving high levels of long-term CAR expression, non-viral approaches provide improved safety and scalability but currently face challenges in efficiency and persistence, particularly regarding sustained therapeutic effects in patients [72, 73]. Ongoing studies are focused on enhancing the efficiency and durability of non-viral delivery systems to improve gene transfer performance and therapeutic persistence. The optimization of these approaches is directed towards improving safety, reproducibility, and clinical applicability across diverse patient populations.

## Combination strategies to overcome tumor microenvironment–mediated resistance in CAR-T therapy

One strategy to enhance CAR-T cell therapeutic efficacy involves combining it with other treatment modalities. Given the immunosuppressive effects of myeloid-derived suppressor cells (MDSCs) on T-cell function, integrating CAR-T cells with agents that deplete MDSCs may augment therapeutic efficacy [79, 80]. This combinatorial approach to improving CAR-T cell function in B-cell malignancies may also extend to acute myeloid leukemia (AML) and includes strategies such as interleukin-15 (IL-15) upregulation, interleukin-18 (IL-18)–based T cells redirected for universal cytokine-mediated killing (TRUCKs), and supplementation with interleukin-7 (IL-7), IL-15, and IL-21 during *ex vivo* expansion [81, 82].

Checkpoint blockade using anti-programmed cell death protein 1 (PD-1) or anti-cytotoxic T-lymphocyte-associated protein 4 (CTLA-4) antibodies has been investigated to mitigate CAR-T cell exhaustion, particularly in tumors with low immunogenicity, such as AML; however, clinical efficacy remains variable and may be constrained by complex immunosuppressive signaling networks [83]. Additionally, combining CAR-T cells with apoptosis-regulating drugs, including B-cell lymphoma 2 (BCL-2) inhibitors, cytoreductive chemotherapy, or hypomethylating agents like azacitidine or decitabine, may decrease tumor burden and improve the tumor microenvironment [84]. Immunomodulatory and epigenetic strategies, such as lenalidomide treatment and TGF-β inhibition, have also demonstrated potential in enhancing CAR-T cell activity against cancer cells [85–88]. Metabolic modulation, including L-arginine enhancement, kynureninase overexpression, and indoleamine 2,3-dioxygenase 1 (IDO1) inhibition, represents another emerging strategy, as tumor microenvironment–induced hypoxia and nutrient deprivation impair CAR-T cell function; these methods may improve CAR-T cell persistence and cytotoxicity [89].

## Tumor cell killing mechanism of CAR-T cells

CAR-T cell therapies are engineered to target tumor-specific or tumor-associated antigens that are highly expressed on the surface of cancer cells [88, 89]. CAR-T cells recognize and bind to specific protein or glycoprotein antigens that are upregulated on the surface of cancer cells. The scFv component tightly binds to the target antigen, facilitating specific recognition by CAR-T cells [90]. Similar to conventional T-cell activation, binding to the antigen activates intracellular signaling domains within the CAR structure. Following antigen recognition by the tumor antigen-binding moiety, signaling domains, such as CD3^ζ^, are activated, initiating intracellular signaling cascades. Unlike conventional T cells, CAR-T cells operate independently of major histocompatibility complex (MHC) restrictions and incorporate co-stimulatory domains, resulting in enhanced activation and robust antitumor responses, although this can also complicate their management [91]. Activated CAR-T cells eliminate cancer cells through multiple mechanisms, including direct cytotoxic molecule release, cytokine secretion (such as interferon-γ and tumor necrosis factor-α), and recruitment of other immune cells. These mechanisms culminate in tumor cell lysis and apoptosis [92, 93].

## Clinical success in hematological malignancies

CAR-T cell therapy has demonstrated significant efficacy in treating hematological malignancies. Notably, CARs targeting the CD19 antigen have shown strong therapeutic efficacy by specifically targeting and eliminating CD19^+^ leukemia cells [94–96]. This approach has also been applied successfully in treating relapsed/refractory B-cell non-Hodgkin’s lymphoma (B-NHL), B-cell acute lymphoblastic leukemia (B-ALL), and chronic lymphocytic leukemia [97–99]. Approximately 60% of patients with B-NHL achieve durable remission and prolonged survival, while nearly 80%–90% of patients with B-ALL attain durable or complete remission after receiving CAR-T cell therapy [99]. Consequently, the efficacy of CAR-T cells in relapsed or refractory B-cell malignancies represents a robust clinical validation of adoptive cell therapy [100].

The U.S. FDA has approved several CAR-T cell therapies, including Kymriah^®^ , Yescarta^®^ , Tecartus^®^ , and lisocabtagene maraleucel (Breyanzi^®^ ), for various hematologic malignancies. Kymriah^®^ , a second-generation CAR-T cell therapy targeting the B-cell antigen CD19 for children and young adults with ALL, was the first CAR-T cell therapy to receive approval from both the FDA and the European Medicines Agency (EMA) [101, 102]. Numerous clinical trials are currently underway, evaluating these CAR-T cells for additional indications [101, 103].

The success of CAR-T therapy can be attributed to a combination of favorable factors. The CD19 antigen is abundantly and uniformly expressed on the surface of B-cell tumors, providing a clear and consistent therapeutic target [104, 105]. Long-term follow-up studies continue to elucidate the mechanisms behind durable remissions. Notably, a landmark single-cell atlas of CAR-T cells from a patient in remission for eight years revealed that a population of CD4^+^ T cells with a T helper 2-like phenotype played a crucial role in long-term control, offering valuable insights for designing therapies aimed at achieving lasting cures [106].

## CAR-T cell therapy for solid tumors

CAR-T cell therapy has achieved significant success in treating hematological malignancies [94–98]. Nonetheless, challenges such as tumor heterogeneity, inadequate trafficking, and T-cell dysfunction—driven by factors within the TME—have constrained its effectiveness against solid tumors [106, 107]. Despite these obstacles, CAR-T cell therapy for solid tumors is under active investigation, with recent clinical trials indicating promising safety and efficacy [106, 108–110]. Notably, recent trials have demonstrated the effectiveness of locally administered CAR-T cell therapy for aggressive malignant gliomas. In a phase 1 study involving 65 patients with recurrent high-grade glioma, 50% of the 58 patients receiving at least three CAR-T cell infusions exhibited stable disease or better outcomes, which included two partial responses and two complete responses. While no dose-limiting toxicities were reported, 35% of patients experienced grade 3 or higher toxicities potentially related to CAR-T therapy [108].

In another phase 1 clinical trial involving six patients with recurrent glioblastoma (GBM), intrathecal administration of bivalent CAR-T cells targeting both epidermal growth factor receptor (EGFR) and IL13Rα2 resulted in tumor size reduction in all patients within 24–48 h post-administration, although one patient experienced side effects such as fatigue and weakness [109]. Similarly, a phase 1 trial involving three patients with recurrent GBM treated with intraventricular CARv3-TEAM-E T cells demonstrated rapid tumor regression in all cases, with a durable response lasting over 150 days in one patient, without grade ≥3 adverse events or dose-limiting toxicities [109].

CAR-T cells have also shown favorable outcomes in gastrointestinal cancers, with Claudin18.2 (CLDN18.2)-targeted cells achieving an objective response rate (ORR) of approximately 30%–40%. Some studies utilizing CLDN18.2 CAR-T cells reported no instances of grade ≥3 CRS, immune effector cell-associated neurotoxicity syndrome (ICANS), treatment-related fatalities, or dose-limiting toxicities (DLTs) in the study population [111]. A clinical trial combining CAR-T cells with a CAR-T cell-amplifying RNA vaccine indicated promising results in claudin 6 (CLDN6)-positive solid tumors, with an unconfirmed ORR of 33%, including one complete response and one case of grade 3 CRS [112]. These findings reflect progress in the application of CAR-T cell therapies for solid tumors.

However, the encouraging efficacy observed in preclinical mouse models has not consistently translated to clinical success. Therapeutic responses in solid tumors are limited by intrinsic tumor properties and microenvironmental barriers, including antigenic heterogeneity, immune escape mechanisms, and impaired CAR-T cell proliferation, infiltration, persistence, and survival within the TME [113]. While early clinical evidence, including prostate-specific membrane antigen (PSMA)-targeted CAR-T strategies, suggests potential anti-tumor activity in selected malignancies, overall clinical advancement remains modest. Importantly, no CAR-T cell therapy has been approved by the FDA or EMA for the treatment of solid tumors, highlighting the urgent need for improved antigen targeting, enhanced CAR-T cell persistence, and effective modulation of the tumor microenvironment (Table 1) [114–116].

**Table 1:** CAR-T cell therapy: Clinical outcomes, key challenges, associated toxicities, and strategic directions

**CAR-T platform**	**Cancer type**	**Major findings / clinical outcomes**	**Main challenges / toxicities**	**Emerging strategies / improvements**	**Ref.**
CD19 CAR-T therapy	B-ALL, B-NHL, CLL	High therapeutic efficacy with durable remission in hematological malignancies; ∼60% durable remission in B-NHL and ∼80–90% remission in B-ALL	CRS, neurotoxicity, antigen escape, relapse	Multi-target CARs, safety switches, cytokine modulation	[94–100]
Locoregional CAR-T therapy	Recurrent high-grade glioma	Stable disease or better in 50% of patients receiving repeated infusions	Grade ≥3 toxicities in 35% of patients	Local administration to improve tumor targeting and reduce systemic toxicity	[106, 108]
EGFR/IL13Rα2-targeted CAR-T	Glioblastoma	Rapid tumor reduction observed within 24–48 h after treatment	Fatigue, weakness, limited patient number	Dual-antigen targeting to reduce antigen escape	[109]
CARv3-TEAM-E T cells	Recurrent glioblastoma	Rapid tumor regression and durable response in one patient	Limited long-term follow-up data	Improved persistence and reduced exhaustion	[109]
CLDN18.2-targeted CAR-T	Gastrointestinal cancers	Objective response rate of ∼30–40% with manageable toxicity profile	Antigen heterogeneity and limited persistence	Combination therapy and TME modulation	[111]
CLDN6 CAR-T + RNA vaccine	CLDN6-positive solid tumors	Promising anti-tumor response with an ORR of 33%	Grade 3 CRS reported	RNA vaccine-mediated CAR-T amplification	[112]
PSMA-targeted CAR-T	Prostate cancer	Preliminary anti-tumor activity in selected patients	Poor infiltration and immunosuppressive TME	Logic-gated and armored CAR approaches	[114–116]
UCART19 allogeneic CAR-T	B-ALL	Reduced alloreactivity and encouraging clinical efficacy	CRS, prolonged cytopenia, severe viral infections	TRAC/CD52 knockout and safety monitoring	[124, 129, 149, 153]
CTA101 dual-target CAR-T	CD19/CD20-positive malignancies	60% CR/CRi with sustained MRD negativity	Limited durability assessment	Dual-targeting and genome editing	[130]
WU-CART007	CD7-positive malignancies	MRD-negative CR/CRi and clinical responses observed	Grade ≥3 CRS and limited response duration	TCR disruption and CAR optimization	[130]
CAR-NK therapy (anti-CD19/IL-15/iC9)	NHL, CLL	Reduced CRS and neurotoxicity with a favorable safety profile	Limited persistence and cryopreservation sensitivity	IL-15 incorporation and suicide switches	[129, 134–136]
Logic-gated Tmod CAR	EGFR-positive solid tumors	Enhanced tumor specificity and reduced off-tumor toxicity	Long-term efficacy remains uncertain	HLA-dependent logic-gated activation	[137, 138]
Logic-gated CAR-NK	AML, myelodysplastic syndromes	Reduced systemic toxicity through tumor-specific activation	Early clinical-stage evidence	Tumor-restricted activation systems	[139]
STAR-T platform	Relapsed/refractory B-cell NHL	Reduced exhaustion and improved persistence compared with conventional CAR-T	Limited long-term comparative evidence	Endogenous CD3 signaling integration	[139]
Armored CAR-T (IL-12 secreting)	Solid tumors	Enhanced pro-inflammatory TME modulation and immune recruitment	Risk of excessive inflammatory toxicity	Controlled cytokine-expression systems	[149–152]
Bispecific and tandem CARs	Hematologic and solid tumors	Improved antigen recognition and reduced antigen escape	Structural complexity and exhaustion risk	OR-gate and AND-gate CAR engineering	[138, 139]
CRISPR/Cas9- and TALEN-edited CAR-T	Hematologic and solid malignancies	Reduced GvHD and immune rejection in allogeneic CAR therapies	Off-target editing and genomic instability concerns	Site-specific CAR integration and genome editing optimization	[74, 75, 128, 129]
Safety-switch CAR systems (iC9, tEGFR)	Hematologic malignancies	Improved control of severe CRS and neurotoxicity	Premature CAR elimination may reduce efficacy	Inducible suicide-switch optimization	[136, 137, 155–157]
Solid tumor CAR-T therapy overall	Multiple solid tumors	Encouraging early clinical activity	TME suppression, antigen heterogeneity, poor trafficking, off-tumor toxicity	Multi-target CARs, armored CARs, metabolic modulation, checkpoint blockade	[6, 25, 113, 142, 152]

Abbreviations: AML, acute myeloid leukemia; B-ALL, B-cell acute lymphoblastic leukemia; B-NHL, B-cell non-Hodgkin lymphoma; CAR, chimeric antigen receptor; CAR-NK, chimeric antigen receptor natural killer; CAR-T, chimeric antigen receptor T-cell; CD, cluster of differentiation: CLDN18;2, claudin 18.2; CLDN6, claudin 6; CLL, chronic lymphocytic leukemia; CR, complete response; CRi, complete response with incomplete hematologic recovery; CRISPR/Cas9, clustered regularly interspaced short palindromic repeats/CRISPR-associated protein 9; CRS, cytokine release syndrome; EGFR, epidermal growth factor receptor; GvHD, graft-versus-host disease; HLA, human leukocyte antigen; iC9, inducible caspase 9; IL, interleukin; IL13Rα2, interleukin-13 receptor alpha 2; MRD, minimal residual disease; NHL, non-Hodgkin lymphoma; NK, natural killer; ORR, objective response rate; PSMA, prostate-specific membrane antigen; RNA, ribonucleic acid; STAR, synthetic T-cell receptor and antigen receptor; TALEN, transcription activator-like effector nuclease; TCR, T-cell receptor; tEGFR, truncated epidermal growth factor receptor; TME, tumor microenvironment; TRAC, T-cell receptor alpha constant.

### Challenges and limitations

Despite these promising results, several significant challenges limit the clinical application of CAR-T therapy. In clinical trials, patient participation is often curtailed due to disease progression and cell production failures; reports indicate that 0%–31% of patients may not receive CAR-T infusions [117, 118]. The costs associated with CAR-T cell therapies, which can amount to hundreds of thousands of dollars, also present a barrier to treatment [119]. Moreover, CAR-T cell therapy faces multifaceted challenges, including antigen selection, treatment tolerance and safety, the presence of tumor cells lacking specific antigens or exhibiting heterogeneity, and the potential for tumor cells to develop resistance through downregulation of antigen expression and promotion of immune inhibitory factors. CRS induced by CAR-T cell therapy can manifest as fever, difficulty breathing, hypotension, nausea, and vomiting, posing significant safety concerns [120]. CRS arises from the massive activation and proliferation of CAR-T cells and other immune cells, leading to the excessive release of inflammatory cytokines such as IL-6 and interferon gamma (IFN-γ) [121–123]. CRS has been identified as a common and challenging side effect for immunotherapy recipients. For instance, one study reported that 19 patients (91%) experienced severe grade CRS, while 8 patients (38%) exhibited grade 1–2 neurotoxicity, 2 patients (10%) had grade 1 acute skin GvHD, and 6 patients (32%) presented with grade 4 prolonged cytopenia. Additionally, two treatment-related fatalities were attributed to neutropenic sepsis and pulmonary hemorrhage [124]. Despite these adverse effects, the study also noted that 14 out of 21 patients (67%) achieved either a complete response or a complete response without full hematological recovery within 28 days post-infusion [124].

Clinical consequences of CRS can range from mild fever to life-threatening hypotension and multi-organ failure. Severe CRS can be managed with targeted therapeutic approaches, including the anti-IL-6 receptor antibody tocilizumab [124--126]. Additionally, CAR-T cell therapy can lead to a complex neurological syndrome characterized by confusion, aphasia, tremors, seizures, and even cerebral edema, necessitating management with corticosteroids and supportive care, while ongoing research seeks to better understand and mitigate this serious adverse event. In June 2025, the U.S. Food and Drug Administration eliminated Risk Evaluation and Mitigation Strategies (REMS) requirements for all approved CAR-T therapies, thereby streamlining access while maintaining safety protocols [127].

Concerns have also been raised regarding the risk of viral infections following alemtuzumab treatment; five patients (24%) experienced grade 3 or higher infections from cytomegalovirus, adenovirus, human metapneumovirus, and BK virus. In contrast, no expansion of UCART19 was observed in the four patients at high risk for viral infections who did not receive alemtuzumab [124]. It is crucial to evaluate the safety and efficacy of these therapies in clinical trials, especially given the significant considerations surrounding the use of CD52 knockout and alemtuzumab strategies. A phase 1 clinical trial demonstrated promising feasibility and efficacy, reporting an overall response rate of 55.8%, which increased to 70.8% at the 320 × 10^6^ dose level; however, the interpretation of these results is limited by the small sample size (*n* ═ 43), lack of a control arm, and early-phase design. Safety remains a concern, with 88.0% of patients experiencing grade ≥3 adverse events and 53.5% developing infections (23.3% grade ≥3), despite a low incidence of severe CRS (2.3%) and no reported high-grade neurotoxicity. Furthermore, the short follow-up period restricts the assessment of response durability and long-term safety, emphasizing the need for larger, controlled studies [128, 129].

Several clinical trials have demonstrated the use of transcription activator-like effector nucleases (TALENs) or CRISPR-Cas9 technology to disrupt the endogenous TCR in allogeneic donor-derived TCR-ablated CAR-T cell therapies, including CD22-targeting UCART22, CD19/CD20 dual-targeting CTA101, and CD7-targeting WU-CART007, all of which have shown promising outcomes. Notably, instances of GvHD were not reported in all trials, and the incidence of grade 3 or higher CRS varied, with no cases in CTX130, 16.7% in CTA101, and 31% in WU-CART007 [125, 130]. Furthermore, across all trials, only ≤10% of patients experienced ICANS. In terms of efficacy, UCART22 achieved response rates of 67% at dose level 2 (DL2) and 50% at dose level 3 (DL3), CTA101 demonstrated a 60% rate of complete response (CR) or complete response with incomplete hematological recovery (CRi) with sustained minimal residual disease (MRD) negativity, and WU-CART007 reported a 43% response at DL2, with MRD-negative CR/CRi and a median response duration of 86 days [129, 130].

Other immune cells, such as NK cells and macrophages, exhibit significant anti-cancer effects and do not typically lead to major adverse events associated with GvHD. These innate immune cells have been effectively harnessed for allogeneic cell therapy. However, CAR-NK cells face limitations, including restricted expansion and reduced persistence *in vivo* compared to CAR-T cells. This crucial limitation necessitates additional genetic modifications to improve their efficacy *in vivo*, such as the incorporation of IL-15 [130, 131]. Additionally, CAR-NK cells are sensitive to cryopreservation, necessitating careful storage and timely administration to patients. Nonetheless, allogeneic NK cells derived from cord blood or induced pluripotent stem cells (iPSCs) do not require complete human leukocyte antigen (HLA) matching for safe administration and have demonstrated a favorable safety profile following adoptive immunotherapy in cancer patients [132, 133]. In a phase 1/2 clinical study of CD19-directed CAR-NK cells for patients with relapsed or refractory non-Hodgkin lymphoma (NHL) or chronic lymphocytic leukemia (CLL), the infused CAR-NK product exhibited a CAR transduction efficiency of approximately 49.0% [134, 135]. Despite these encouraging findings, the long-term efficacy, persistence, and safety of CAR-NK therapies remain incompletely understood, necessitating further investigation in larger clinical studies.

### Hurdles in solid tumors

While CAR-T cell therapies have demonstrated efficacy in treating hematologic malignancies, their application in solid tumors is hindered by several challenges, notably on-target off-tumor toxicity. To improve target specificity and reduce adverse effects, logic-gated CARs provide an advanced methodology that confines CAR activity to the tumor microenvironment [136]. One mechanism enhancing this specificity is the dual-receptor logic gate. For instance, the NCT06682793 trial is exploring the Tmod system in EGFR-positive solid tumors with HLA-A02 loss of heterozygosity. This system integrates a stimulator CAR that targets EGFR and a blocker receptor that detects HLA-A02 expression, ensuring that normal cells expressing HLA-A*02 are preserved while malignant cells lacking this marker are selectively activated by the CAR. This method significantly increases the specificity of CARs for tumor cells while concurrently reducing toxicities [137, 138].

In a similar vein, the NCT06325748 trial investigated a logic-gated CAR-NK cell therapy for AML and myelodysplastic syndromes. This study revealed that CAR-NK cells are activated solely in the presence of tumor-specific antigens and activation mechanisms, thereby minimizing systemic toxicity and supporting the potential application of this therapy beyond hematological malignancies [139]. However, CAR-T therapies also face limitations in CAR signaling and are prone to functional exhaustion. To mitigate these issues, the synthetic synthetic T-cell receptor and antigen receptor (STAR) platform has been developed, which integrates an antigen-recognition domain to engage endogenous CD3 signaling pathways. This strategy significantly reduces exhaustion while promoting proliferation and long-term persistence. Clinical investigations indicate that STAR-T cells exhibit reduced toxicity and enhanced efficacy compared to both BB^ζ^CAR-T and 28^ζ^CAR-T cells [139]. The NCT05631912 trial is currently evaluating the clinical application of a CRISPR-Cas9-mediated disruption of TRAC to integrate an anti-CD19-STAR construct in patients with relapsed or refractory B-cell NHL.

Ongoing investigations in this domain are optimizing the clinical application of both autologous and allogeneic CAR cell therapies, facilitating the development of safer and more effective immunotherapies. Future CAR engineering strategies, including bispecific CARs, logic-gated CARs, and STAR constructs, are paving the way for promising alternatives to overcome antigen escape, enhance persistence and proliferation, and reduce exhaustion in allogeneic cell therapy. Despite these innovations, continual assessment is necessary to validate their efficacy and safety. Beyond the aforementioned limitations, including toxicities, translating CAR-T efficacy to solid tumors remains a considerable challenge, primarily due to the immunosuppressive tumor microenvironment characteristic of most solid malignancies [139, 140]. This microenvironment constitutes a complex ecosystem replete with regulatory T cells, myeloid-derived suppressor cells, M2 macrophages, and a variety of inhibitory cytokines, such as TGF-β and IL-10, all of which suppress and exhaust infiltrating T cells.

Additionally, solid tumors frequently lack ideal target antigens, as many tumor-associated antigens are also expressed at low levels on critical healthy tissues, heightening the risk of on-target/off-tumor toxicity that can harm essential organs [141]. Tumors also exhibit significant antigen heterogeneity; under the selective pressure from CAR-T cells, they may downregulate target antigens, leading to immune escape and relapse [142, 143]. Finally, the physical structure of solid tumors poses a barrier, as CAR-T cells may struggle to efficiently migrate to the tumor site and penetrate the dense fibrotic stroma and abnormal vasculature to access cancer cells.

Recent trials targeting human epidermal growth factor receptor 2 (HER2) in sarcomas and disialoganglioside GD2 (GD2) in neuroblastoma underscore ongoing efforts, yet response rates remain inferior to those observed in hematological cancers [144, 145]. Overall, CAR-T cell therapy faces substantial limitations, including treatment-related toxicities, organ failure, challenges associated with the manufacturing and scalability of autologous cells, and issues related to persistence and safety in allogeneic approaches. There is also a pressing need for improved, sustainable T-cell sources—such as iPSC-derived CAR-T cells—which, despite their potential, remain hindered by developmental and functional challenges. Innovative strategies incorporating advanced engineering approaches are essential for optimizing allogeneic CAR-T cell therapies. Current strategies focus on improving CAR design, adjusting dosing regimens, and targeting multiple antigens to enhance efficacy; however, these technical solutions do not entirely eliminate the inherent limitations of the platform [127, 128].

### Novel strategies and future perspectives

In response to these challenges, the field is rapidly evolving, with the development of a new generation of CAR-T cells possessing enhanced capabilities. Therapeutic efficacy is being improved through strategies such as selecting less-differentiated T cells, optimizing manufacturing processes, and employing combination or multi-target approaches to circumvent antigen escape. The application of CAR-T therapy at earlier disease stages, alongside alternative antigen targeting and the development of off-the-shelf allogeneic CAR-T cells, represents significant advancements in the field [129, 145, 146]. Despite these advancements, issues such as immune rejection, immunosuppression, infection risk, long-term persistence, logistical complexity, manufacturing scalability, and durable clinical responses continue to pose challenges that may limit broader clinical accessibility.

To counter antigen escape, bispecific and tandem CARs are being engineered to recognize two distinct tumor antigens simultaneously [147]. These sophisticated receptors can be designed with OR-gate logic, where engagement of either antigen triggers activation, or AND-gate logic, which necessitates the presence of both antigens on a target cell to initiate a full response, thereby enhancing specificity and reducing the likelihood of relapse due to antigen loss [146–148]. Although these multi-targeting approaches show promise, they are not without limitations; their structural and signal transduction complexity may impact CAR manufacturing processes, stability, and exhaustion profiles, raising concerns regarding their transition to clinical use. The heterogeneous antigen expression within solid tumors continues to compromise therapeutic efficacy.

Moreover, to counter the immunosuppressive tumor microenvironment, “armored” CARs are being developed. These fourth-generation constructs are engineered to constitutively or inducibly express protective or stimulatory molecules, such as cytokines, cytokine receptors, or ligands that can block inhibitory signals. For example, CAR-T cells engineered to secrete IL-12 can help repolarize the microenvironment toward a pro-inflammatory state and recruit innate immune cells to the tumor site [149–152]. However, the therapeutic potential of armored CAR constructs may be limited by prolonged, uncontrolled pro-inflammatory cytokine release, which can exacerbate systemic toxicity, particularly in patients with advanced disease. The balance between enhanced immune activation and off-target inflammatory damage remains insufficiently documented in clinical settings.

The integration of gene editing technologies, such as CRISPR-Cas9 and TALEN, holds promise for improving the safety, efficacy, and persistence of allogeneic CAR-T and CAR-NK therapies while limiting immune evasion and enhancing tumor targeting specificity—critical factors for expanding their clinical application in both hematologic and solid malignancies [128, 129]. Nevertheless, concerns regarding off-target genome editing, chromosomal rearrangements, and long-term genomic instability necessitate thorough investigation before widespread clinical implementation. Furthermore, permanent genomic modifications may raise ethical and regulatory concerns that complicate clinical translation and standardization. Cytokine design is particularly promising for enhancing the efficacy of CAR-T and CAR-NK cell therapies by improving survival, proliferation, and overall *in vivo* function. Several clinical trials (NCT04245722, NCT06342986, NCT04623944, NCT05182073, NCT06733935, and NCT03774654) have demonstrated that the co-expression of cytokines such as IL-15 and IL-10 in CAR-NK and CAR-NKT cell products enhances their survival and cytotoxic potential. Overall, cytokine engineering is paving the way for the advancement of next-generation CAR-based immunotherapies [129]. Despite the anticancer benefits and improved cellular persistence associated with cytokine engineering, uncontrolled excessive cytokine activation may lead to immune exhaustion and cytokine-mediated toxicities, necessitating a precisely controlled approach.

Trials NCT05350787, NCT04093596, and NCT05336409 involve knocking out endogenous TCRs to minimize GvHD and immune rejection, thereby enhancing the safety and efficacy of allogeneic CAR-T therapies. TALEN-based genome editing is also being employed in UCART19, where TRAC and CD52 gene knockouts enhance persistence and reduce alloreactivity in patients with B-ALL [129]. The persistence and adverse reactivity in B-ALL patients are improved through TRAC and CD52 gene knockouts using TALEN-based genome editing in UCART19 [149, 153]. Similarly, the use of CRISPR-Cas9 to disrupt TCR and HLA reduces immune rejection and GvHD, as demonstrated by the ALLO-715 assessment in the NCT04093596 trial [150]. Despite these positive outcomes, the long-term implications of immune system complications, such as secondary malignancy risk, altered immune surveillance, and prolonged immunodeficiency due to extensive genome editing in immune effector cells, remain poorly understood. Additionally, CRISPR-Cas9-mediated CAR knock-in at the TRAC locus is under investigation in several trials (NCT06680037, NCT05757700, NCT03666000, NCT04629729) to evaluate CAR expression and minimize alloreactivity, with consistent outcomes highlighting the pivotal role of gene editing in enhancing the success of allogeneic CAR-based immunotherapies. However, variability in editing efficiency, transgene integration, and cellular fitness may affect therapeutic reproducibility across diverse patient populations.

**Figure 3.**
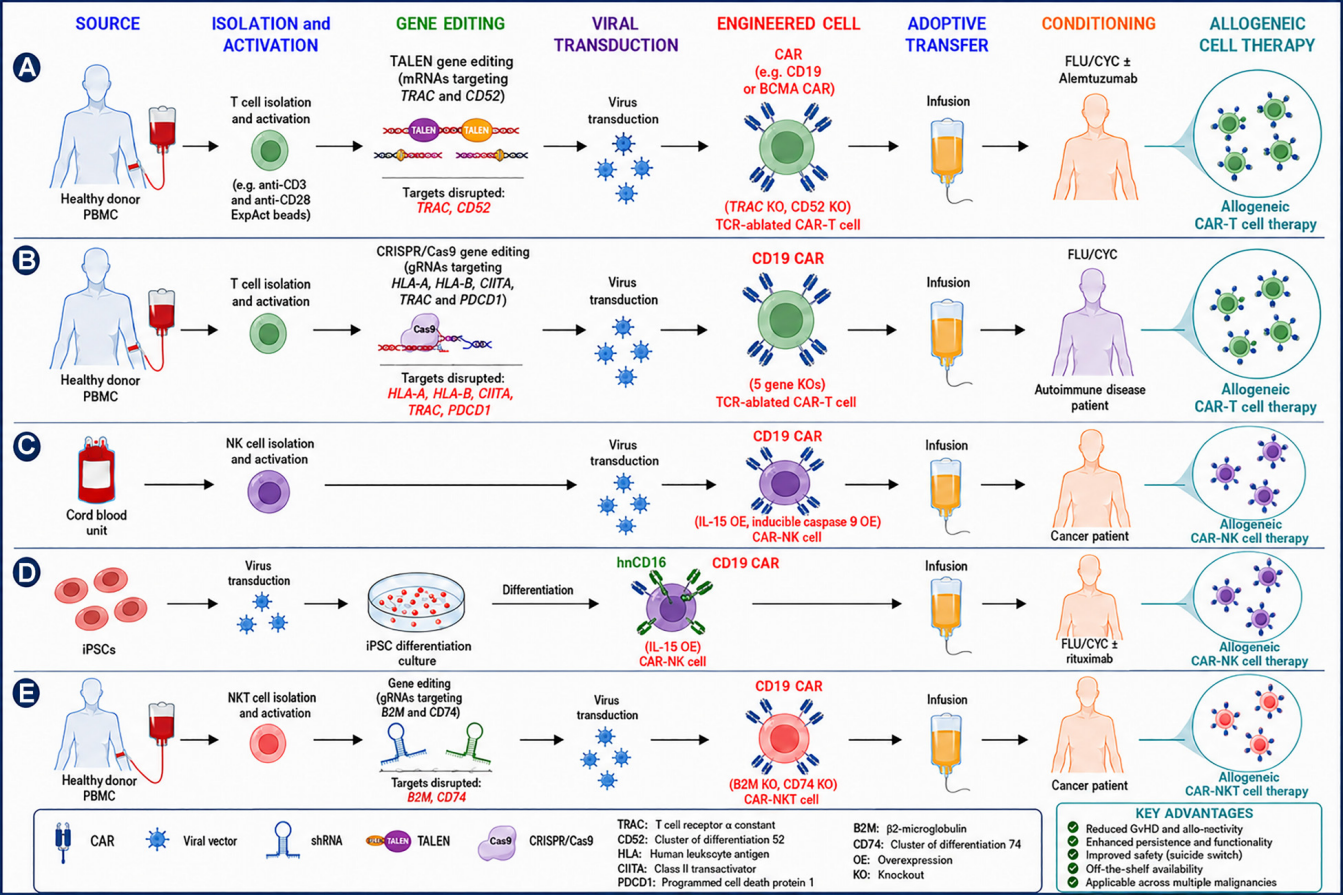
**Advanced engineering strategies in allogeneic CAR-based immunotherapies.** (A) Healthy donor PBMC-derived T cells are isolated, activated, edited with TALEN to disrupt TRAC and CD52, transduced with CAR constructs (e.g., CD19 or BCMA), and infused after FLU/CYC ± alemtuzumab conditioning to generate allogeneic CAR-T products for cancer. (B) Healthy donor PBMC-derived T cells are edited with CRISPR/Cas9 to disrupt HLA-A, HLA-B, CIITA, TRAC, and PDCD1, then transduced with a CD19 CAR and infused after FLU/CYC conditioning as an allogeneic CAR-T strategy for autoimmune disease. (C) Cord blood-derived NK cells are isolated, activated, virally transduced with a CD19 CAR, and further engineered with IL-15 and a safety-switch module to produce allogeneic CAR-NK cells for cancer therapy. (D) iPSC-derived NK cells are generated through viral engineering and directed differentiation, yielding an off-the-shelf CAR-NK platform with enhanced functional support for adoptive transfer. (E) Healthy donor PBMC-derived NKT cells are isolated, activated, edited to disrupt B2M and CD74, transduced with a CD19 CAR, and administered as allogeneic CAR-NKT therapy. Overall, the figure highlights how gene editing, cytokine support, and safety-switch engineering are being used to reduce alloreactivity and GvHD, improve persistence and safety, and expand the feasibility of off-the-shelf CAR-based immunotherapies. Adapted from Li et al., 2025 [129]. Abbreviations: B2M, beta-2 microglobulin; BCMA, B-cell maturation antigen; CAR, chimeric antigen receptor; CAR-NK, chimeric antigen receptor natural killer; CAR-NKT, chimeric antigen receptor natural killer T-cell; CAR-T, chimeric antigen receptor T-cell; CD19, cluster of differentiation 19; CD52, cluster of differentiation 52; CD74, cluster of differentiation 74; CIITA, class II transactivator; CRISPR/Cas9, clustered regularly interspaced short palindromic repeats/CRISPR-associated protein 9; CYC, cyclophosphamide; FLU, fludarabine; GvHD, graft-versus-host disease; HLA, human leukocyte antigen; IL-15, interleukin-15; iPSC, induced pluripotent stem cell; NK, natural killer; NKT, natural killer T-cell; PBMC, peripheral blood mononuclear cell; PDCD1, programmed cell death protein 1; TALEN, transcription activator-like effector nuclease; TCR, T-cell receptor; TRAC, T-cell receptor alpha constant.

The CAR-NK cells engineered with anti-CD19 CAR, IL-15, and inducible caspase 9 (iC9) have shown improved safety and a reduction in CRS and neurotoxicity, as evidenced by the NCT03056339 clinical trial [129, 136]. Inducible suicide switches, particularly the iC9 system, are integrated into CAR constructs to facilitate the controlled elimination of engineered immune cells, thereby minimizing severe toxicity risks such as CRS and neurotoxicity. The NCT03056339 trial indicated that CAR-NK cells featuring anti-CD19 CAR, IL-15, and iC9 enhanced safety by mitigating these risks. Furthermore, a truncated epidermal growth factor receptor (tEGFR) switch in an IL-15-expressing CAR-NK cell product improves safety for relapsed or refractory CD19-positive B-cell malignancies (NCT05336409) by enabling selective cell depletion when necessary [137]. Despite promising outcomes from suicide switch technologies that significantly enhance controllability and safety, the premature elimination of therapeutic cells may restrict sustained anti-tumor efficacy and diminish long-term remission durability. Additionally, the clinical thresholds for activating these safety switches remain inadequately standardized. The CD38 knockout in donor-derived CAR NK cells represents an innovative approach to prevent antibody-mediated fratricide; the elimination of CD38 enhances NK cell survival and functionality, thereby improving therapeutic efficacy [152]. However, the broader immunological implications of CD38 deletion on NK cell biology and host immune regulation warrant further mechanistic exploration. The integration of bispecific T cell engager technology and gene editing, including PD-1 knockout, into CAR platforms further amplifies anti-tumor efficacy by recruiting bystander T cells and overcoming tumor-induced exhaustion, thereby enhancing persistence and cytotoxic function [153]. Nevertheless, prolonged checkpoint disruption may lead to increased immunological complications, including severe inflammatory responses. These innovative gene editing and cytokine modification strategies in CAR-T and CAR-NK therapies are advancing immunotherapy by addressing critical limitations related to toxicity, safety, persistence, and efficacy, thus reshaping their broader clinical applications. Despite significant technological advancements in CAR therapies, many next-generation CAR platforms remain in early-phase clinical evaluation, and robust long-term comparative studies are still lacking.

### The quest for “off-the-shelf” products and safety switches

Current research is increasingly focused on the development of “off-the-shelf” or allogeneic CAR-T products [38, 39, 151]. These therapies, derived from the T cells of healthy donors, aim to provide readily available, cost-effective, and standardized treatments, promising greater accessibility and affordability for a broader patient population while overcoming the logistical and financial burdens associated with personalized autologous manufacturing. This platform is particularly crucial for patients with rapidly progressive diseases or insufficient autologous T-cell availability for CAR-T manufacturing [129]. In addition to the well-known CAR-T cells, other CAR-engineered immune cell types, including macrophages, NK cells, invariant natural killer T (NKT) cells, and gamma delta T (γ δ T) cells, are being extensively investigated in multiple trials. Among these, CAR-NK and CAR-NKT cell therapies have shown promising results in clinical trials [134–136]. However, the relatively limited *in vivo* persistence and expansion capacity of CAR-NK and CAR-NKT cells compared to conventional CAR-T cells may hinder their durable therapeutic roles and effective anti-tumor potential. Several clinical trials have reported favorable outcomes for CAR-NK and CAR-NKT cell therapies, particularly regarding reduced GvHD and host-mediated allorejection. Healthy donor peripheral blood mononuclear cells (PBMCs), cord blood, and stem cells, including iPSCs and hematopoietic stem and progenitor cells (HSPCs), are among the selected sources for generating these allogeneic CAR cell therapies [132].

However, allogeneic CAR cell therapies may be compromised by host cell-mediated allorejection, as differences in MHC molecules can lead to donor cells being recognized as foreign. If host immune cells completely reject the CAR product, the therapeutic benefits may be significantly diminished, as malignant cells are not effectively eliminated. To address this challenge in immunotherapy, various mitigation strategies have been developed, including careful matching of donor and recipient MHC molecules, genetic modification approaches to ablate MHC class I and II molecules, and the administration of immunosuppressive agents. Recent technologies are now attempting to tackle these issues without the need for immunosuppressive agents or donor-recipient matching through advanced gene-editing technologies like CRISPR/Cas9. This approach disrupts genes encoding endogenous T-cell receptors and MHC molecules to create universal CAR-T cells that can be administered to any patient [128, 154]. Nonetheless, concerns regarding off-target mutations, chromosomal abnormalities, genomic instability, and long-term safety monitoring persist. To enhance the safety profile of these potent therapies, researchers are incorporating inducible safety switches [155].

To improve the safety profile, genes encoding proteins such as inducible caspase 9 can be activated by small-molecule drugs, resulting in rapid and selective apoptosis of the CAR-T cell population during severe adverse reactions. This provides a crucial and controllable safety net for both patients and clinicians [156, 157]. Various CAR targets are actively being developed to address other hematological cancers, such as acute myeloid leukemia and non-Hodgkin lymphoma, as well as solid tumors including hepatocellular carcinoma, lung cancer, and ovarian cancer (Figure 3) [158–161]. Despite the expanding range of CAR targets, challenges such as heterogeneous antigen expression, antigen escape, and the immunosuppressive tumor microenvironment continue to hinder therapeutic efficacy in many solid tumors. Addressing these challenges requires improved immune engineering, standardized manufacturing platforms, the development of universally compatible CAR platforms capable of maintaining durable anti-tumor activity with minimal toxicity across diverse patients, and clearer regulatory frameworks to fully realize the clinical potential of allogeneic CAR therapies.

## Conclusion

CAR-T cell therapy has achieved significant clinical success in treating hematological malignancies. However, its efficacy in solid tumors remains limited due to issues such as tumor heterogeneity, poor trafficking, and T-cell dysfunction driven by the immunosuppressive tumor microenvironment. Despite their therapeutic promise, CAR-T cell therapies encounter substantial challenges, including treatment-related toxicities, organ failure, manufacturing and scalability limitations of autologous products, limited persistence, and graft-versus-host disease risk in allogeneic approaches, as well as the need for more sustainable T-cell sources. Advances in genetic engineering, tumor immunology, and clinical strategies are anticipated to enhance the safety, efficacy, persistence, and accessibility of CAR-based therapies. Nevertheless, many next-generation CAR platforms are still in early-phase clinical evaluation, and robust long-term comparative studies are lacking. Future research should thus concentrate on not only enhancing anti-tumor efficacy but also improving safety, manufacturing feasibility, accessibility, and durable clinical benefits across a range of malignancies.

**Conflicts of interest**: Authors declare no conflicts of interest.

**Funding**: Authors received no specific funding for this work.

**AI declaration statement:** AI assistance was used for figure preparation and language improvement to enhance readability.

## References

[1] Bray F, Laversanne M, Sung H, Ferlay J, Siegel RL, Soerjomataram I (2024). Global cancer statistics 2022:GLOBOCAN estimates of incidence and mortality worldwide for 36 cancers in 185 countries. CA Cancer J Clin.

[2] Kaur R, Bhardwaj A, Gupta S (2023). Cancer treatment therapies: traditional to modern approaches to combat cancers. Mol Biol Rep.

[3] Wang GR, Cao LM, Xu ZY, Liu B, Wu Q, Bu LL.

[4] Eckman N, Nejatfard A, Cavet R, Grosskopf AK, Appel EA (2024). Biomaterials to enhance adoptive cell therapy. Nat Rev Bioeng.

[5] BV P (2026). Prospect of chimeric antigen receptor T-cell therapy for cancer. Mol Biol Rep.

[6] Liu C, Wang Z, Zhang W, Cheng G, Cheng S, Qin L (2026). NKG2D CAR-T cells for solid tumor immunotherapy: advances, challenges, and future directions. Front Immunol.

[7] Brudno JN, Maus MV, Hinrichs CS (2024). CAR T cells and T-cell therapies for cancer: a translational science review. JAMA.

[8] Gross G, Waks T, Eshhar Z (1989). Expression of immunoglobulin-T-cell receptor chimeric molecules as functional receptors with antibody-type specificity. Proc Natl Acad Sci U S A.

[9] June CH, Sadelain M (2018). Chimeric antigen receptor therapy. N Engl J Med.

[10] Martinez M, Moon EK (2019). CAR T cells for solid tumors: new strategies for finding, infiltrating, and surviving in the tumor microenvironment. Front Immunol.

[11] Sioud M, Casey NP (2026). Engineering immunity: current progress and future directions of CAR-T cell therapy. Int J Mol Sci.

[12] Zhang B, Yang M, Zhang W, Liu N, Wang D, Jing L (2024). Chimeric antigen receptor-based natural killer cell immunotherapy in cancer: from bench to bedside. Cell Death Dis.

[13] Lui Y, Ferreira Fernandes J, Vuong MT, Sharma S, Santos AM, Davis SJ (2025). The structural biology of T-cell antigen detection at close contacts. Immunol Rev.

[14] Bernard G, Evgin L (2025). Non-signaling but all important: how the linker, hinge, and transmembrane domains in the CAR hold it all together. Front Immunol.

[15] Gómez-Melero S, Hassouneh F, Vallejo-Bermúdez IM, Agüera-Morales E, Solana R, Caballero-Villarraso J (2025). Tandem CAR-T cell therapy: recent advances and current challenges. Front Immunol.

[16] Dreyzin A, Gava F, Lamplugh C, Ma J, Silbert SK (2026). The evolving landscape of CAR T-cell therapy in children and young adults with B-cell acute lymphoblastic leukemia. Mol Ther Oncolytics.

[17] Ismail FS, Gallus M, Meuth SG, Okada H, Hartung HP, Melzer N (2025). Current and future roles of chimeric antigen receptor T-cell therapy in neurology: a review. JAMA Neurol.

[18] Alsaieedi AA, Zaher KA (2025). Tracing the development of CAR-T cell design: from concept to next-generation platforms. Front Immunol.

[19] Chen Z, Zhou X (2025). Decoding signaling architectures: CAR versus TCR dynamics in solid tumor immunotherapy. Acta Biochim Biophys Sin.

[20] Bot A, Scharenberg A, Friedman K, Guey L, Hofmeister R, Andorko JI (2025). In vivo chimeric antigen receptor (CAR)-T cell therapy. Nat Rev Drug Discov.

[21] Fischer-Riepe L, Kailayangiri S, Zimmermann K, Pfeifer R, Aigner M, Altvater B (2024). Preclinical development of CAR T cells with antigen-inducible IL18 enforcement to treat GD2-positive solid cancers. Clin Cancer Res.

[22] Wu Y, Li YR (2026). Frontiers of cytokine engineering in CAR cell therapy for cancer. Front Oncol.

[23] Tang L, Pan S, Wei X, Xu X, Wei Q (2023). Arming CAR-T cells with cytokines and more: innovations in the fourth-generation CAR-T development. Mol Ther.

[24] Hiltensperger M, Krackhardt AM (2023). Current and future concepts for the generation and application of genetically engineered CAR-T and TCR-T cells. Front Immunol.

[25] Hou Y, Hu S, Liu C, Chen X, Wang Y, Li Y (2025). Beyond CAR-T cells: exploring CAR-NK, CAR-M, and CAR-γ δ T strategies in solid tumor immunotherapy. Front Immunol.

[26] Prasad K, Cross RS, Jenkins MR (2026). Progress in the development of cytokine armoured CAR T cells. Nat Rev Immunol.

[27] Kim S, Heo SH, Baek H, Hwang SS (2025). Cytokine engineering in CAR-T cell therapy: next-generation strategies. Immune Netw.

[28] Li Y, Zuo B, Xing Y, Zhang R, Jia L, Yan B (2025). Advances in CAR-T cell therapy for solid tumors. Immune Discov.

[29] Rossari F, Alvisi G, Cusimano M, Beretta S, Birocchi F, Ambrosecchia DI (2025). A cross-talk established by tumor-targeted cytokines rescues CAR T cell activity and engages host T cells against glioblastoma in mice. Sci Transl Med.

[30] Li D, Li X, Zhou WL, Huang Y, Liang X, Jiang L (2019). Genetically engineered T cells for cancer immunotherapy. Signal Transduct Target Ther.

[31] Cao L, Liu Y, Lin G (2025). Strategies for altering delivery technologies to optimize CAR therapy. Int J Mol Sci.

[32] Sun D, Shi X, Li S, Wang X, Yang X, Wan M (2024). CAR T cell therapy: a breakthrough in traditional cancer treatment strategies. Mol Med Rep.

[33] Hamieh M, Mansilla-Soto J, Rivière I, Sadelain M (2023). Programming CAR T cell tumor recognition: tuned antigen sensing and logic gating. Cancer Discov.

[34] Zhang C, Zhuang Q, Liu J, Liu X (2022). Synthetic biology in chimeric antigen receptor T (CAR T) cell engineering. ACS Synth Biol.

[35] Zheng N, Fang J, Xue G, Wang Z, Li X, Zhou M (2022). Induction of tumor cell autosis by myxoma virus-infected CAR-T and TCR-T cells to overcome primary and acquired resistance. Cancer Cell.

[36] Ferreri CJ, Bhutani M (2024). Mechanisms and management of CAR T toxicity. Front Oncol.

[37] Li YR, Fang Y, Niu S, Chen Y, Lyu Z, Yang L (2025). Managing allorejection in off-the-shelf CAR-engineered cell therapies. Mol Ther.

[38] Depil S, Duchateau P, Grupp SA, Mufti G, Poirot L (2020). Off-the-shelf allogeneic CAR T cells: development and challenges. Nat Rev Drug Discov.

[39] Lyu Z, Niu S, Fang Y, Chen Y, Li YR, Yang L (2025). Addressing graft-versus-host disease in allogeneic cell-based immunotherapy for cancer. Exp Hematol Oncol.

[40] Lonez C, Breman E (2024). Allogeneic CAR-T therapy technologies: has the promise been met?. Cells.

[41] Malard F, Peric Z, Ruggeri A, Gedde-Dahl T, Bazarbachi A, Ciceri F (2026). Chronic graft-versus-host disease: current situation and unmet needs—a European position statement. Bone Marrow Transplant.

[42] Kheirolomoom A, Kare AJ, Ingham ES, Paulmurugan R, Robinson ER, Baikoghli M (2022). In situ T-cell transfection by anti-CD3-conjugated lipid nanoparticles leads to T-cell activation, migration, and phenotypic shift. Biomaterials.

[43] Michels KR, Sheih A, Hernandez SA, Brandes AH, Parrilla D, Irwin B (2023). Preclinical proof of concept for VivoVec, a lentiviral-based platform for in vivo CAR T-cell engineering. J Immunother Cancer.

[44] Mukalel AJ, Tylek T, O’Brien E, Frazee C, Geisler HC, Li J (2026). Bioinspired oxidized mRNA lipid nanoparticles for ex vivo engineering of chimeric antigen receptor macrophages targeting solid tumors. Bioeng Transl Med.

[45] Li YR, Zhu Y, Halladay T, Yang L (2025). In vivo CAR engineering for immunotherapy. Nat Rev Immunol.

[46] Volta L, Gill SI (2026). In vivo generation of CAR T cells: biology, delivery platforms, clinical promise, and translational challenges. Blood Immunol Cell Ther.

[47] Sciuto L, Kooijmans SA, Schiffelers RM (2026). Toward in vivo CAR T cell therapy: perfecting CAR and lipid nanoparticle design. Nano Lett.

[48] Sui Y, Hou X, Zhang J, Hong X, Wang H, Xiao Y (2025). Lipid nanoparticle-mediated targeted mRNA delivery and its application in cancer therapy. J Mater Chem B.

[49] Pfeiffer A, Thalheimer FB, Hartmann S, Frank AM, Bender RR, Danisch S (2018). In vivo generation of human CD19-CAR T cells results in B-cell depletion and signs of cytokine release syndrome. EMBO Mol Med.

[50] Agarwal S, Hanauer JD, Frank AM, Riechert V, Thalheimer FB, Buchholz CJ (2020). In vivo generation of CAR T cells selectively in human CD4+ lymphocytes. Mol Ther.

[51] Pentimalli TM, Karaiskos N, Rajewsky N (2025). Challenges and opportunities in the clinical translation of high-resolution spatial transcriptomics. Annu Rev Pathol.

[52] Mantovani A, Allavena P, Marchesi F, Garlanda C (2022). Macrophages as tools and targets in cancer therapy. Nat Rev Drug Discov.

[53] Chou HC, Chiu SJ, Hu TM (2024). Quantitative analysis of macrophage uptake and retention of fluorescent organosilica nanoparticles: implications for nanoparticle delivery and therapeutics. ACS Appl Nano Mater.

[54] Lin ZP, Nguyen LN, Ouyang B, MacMillan P, Ngai J, Kingston BR (2022). Macrophages actively transport nanoparticles in tumors after extravasation. ACS Nano.

[55] Huang M, Yu X, Jiang Z, Li X, Yang S, Luo S (2026). Genetically engineered macrophages delivering TRAIL targeting the Wnt/β-catenin pathway to induce cytotoxicity against TNBC. Cell Commun Signal.

[56] Wang S, Wang M, Yao W (2026). Emerging chimeric antigen receptor-immune cell therapy for pancreatic cancer: mechanisms, clinical advances, and future perspectives. Oncol Res.

[57] Short L, Holt RA, Cullis PR, Evgin L (2024). Direct in vivo CAR T cell engineering. Trends Pharmacol Sci.

[58] Baek Y, Seo YR, Kim S, Jung S, Kim CH, Lee YH (2026). From ex vivo to in vivo: advances in lentiviral vector engineering for CAR-T therapy. Immune Netw.

[59] Watanabe N, Mo F, McKenna MK (2022). Impact of manufacturing procedures on CAR T cell functionality. Front Immunol.

[60] Pinto E, Lione L, Compagnone M, Paccagnella M, Salvatori E, Greco M (2025). From ex vivo to in vivo chimeric antigen T cells manufacturing: new horizons for CAR T-cell based therapy. J Transl Med.

[61] Silveira CR, Corveloni AC, Caruso SR, Macêdo NA, Brussolo NM, Haddad F (2022). Cytokines as an important player in the context of CAR-T cell therapy for cancer: their role in tumor immunomodulation, manufacture, and clinical implications. Front Immunol.

[62] Makni-Maalej K, Alotaibi SM, Fernandes Q, Ahmed SO, Mestiri S, Bougarn S (2026). Mechanistic basis and therapeutic modulation of T cell fitness to enhance CAR-T cell efficacy in hematological malignancies. Front Immunol.

[63] Kowalczyk A, Zarychta J, Marszołek A, Zawitkowska J, Lejman M (2024). Chimeric antigen receptor T cell and chimeric antigen receptor NK cell therapy in pediatric and adult high-grade glioma—recent advances. Cancers (Basel).

[64] Yin H, Wei X (2025). The design of retroviral vectors used in the CAR-T products, risk management, and future perspective. MedComm.

[65] Xin T, Cheng L, Zhou C, Zhao Y, Hu Z, Wu X (2022). In-vivo induced CAR-T cell for the potential breakthrough to overcome the barriers of current CAR-T cell therapy. Front Oncol.

[66] Watanabe N, McKenna MK (2022). Generation of CAR T-cells using gamma retroviral vector. Methods Cell Biol.

[67] Poletti V, Mavilio F (2021). Designing lentiviral vectors for gene therapy of genetic diseases. Viruses.

[68] Giommetti A, Papanikolaou E (2024). Advancements in hematopoietic stem cell gene therapy: a journey of progress for viral transduction. Cells.

[69] Hamilton MP, Sugio T, Noordenbos T, Shi S, Bulterys PL, Liu CL (2024). Risk of second malignancies and T-cell lymphoma after chimeric antigen receptor T-cell therapy. N Engl J Med.

[70] Hu J, Dunbar CE (2024). T-cell lymphomas in recipients of CAR-T cells: assessing risks and causalities. Blood.

[71] Mironova KA, Zotova AD, Deyev IE (2026). Adenosine pathway of T cell regulation with a chimeric antigen receptor. Russ J Bioorg Chem.

[72] Albalawi YA (2025). Applications of nanoparticles in CAR-T cell therapy: non-viral manufacturing, enhancing in vivo function, and in vivo generation of CAR-T cells. Med Oncol.

[73] Tretbar US, Rurik JG, Rustad EH, Sueruen D, Koehl U, Olweus J (2024). Non-viral vectors for chimeric antigen receptor immunotherapy. Nat Rev Methods Primers.

[74] Shams F, Sharif E, Abbasi-Kenarsari H, Hashemi N, Hosseini MS, Heidari N (2025). CRISPR/Cas9 technology for modifying immune checkpoint in CAR-T cell therapy for hematopoietic malignancies. Curr Gene Ther.

[75] Eyquem J, Mansilla-Soto J, Giavridis T, van der Stegen SJC, Hamieh M, Cunanan KM (2017). Targeting a CAR to the TRAC locus with CRISPR/Cas9 enhances tumour rejection. Nature.

[76] Chen J, Qiu S, Li W, Wang K, Zhang Y, Yang H (2023). Tuning charge density of chimeric antigen receptor optimizes tonic signaling and CAR-T cell fitness. Cell Res.

[77] Zhang J, Hu Y, Yang J, Li W, Zhang M, Wang Q (2022). Non-viral, specifically targeted CAR-T cells achieve high safety and efficacy in B-NHL. Nature.

[78] Dingfelder J, Taubmann J, von Heydebrand F, Aigner M, Bergmann C, Knitza J (2025). Exploring CAR T-cell dynamics: balancing potent cytotoxicity and controlled inflammation in CAR T-cells derived from systemic sclerosis and myositis patients. Int J Mol Sci.

[79] Balke-Want H, Keerthi V, Cadinanos-Garai A, Fowler C, Gkitsas N, Brown AK (2023). Non-viral chimeric antigen receptor (CAR) T cells going viral. Immuno-Oncol Technol.

[80] Liu Z, Zhou Z, Dang Q, Xu H, Lv J, Li H (2022). Immunosuppression in tumor immune microenvironment and its optimization from CAR-T cell therapy. Theranostics.

[81] Tomai R, De Las Rivas J, Fetica B, Bergantim R, Filipic B, Gagic Z (2025). Challenges in the preclinical design and assessment of CAR-T cells. Front Immunol.

[82] Su X, Li J, Xu X, Ye Y, Wang C, Pang G (2024). Strategies to enhance the therapeutic efficacy of anti-PD-1 antibody, anti-PD-L1 antibody and anti-CTLA-4 antibody in cancer therapy. J Transl Med.

[83] Rossetti R, Brand H, Lima SC, Furtado IP, Silveira RM, Fantacini DM (2022). Combination of genetically engineered T cells and immune checkpoint blockade for the treatment of cancer. Immunother Adv.

[84] Zhang Z, Su M, Jiang P, Wang X, Tong X, Wu G (2024). Unlocking apoptotic pathways: overcoming tumor resistance in CAR-T-cell therapy. Cancer Med.

[85] Greiner J, Schuler PJ, Schrezenmeier H, Weiss J, Bulach C, Goetz M (2026). Different immunotherapeutic combinations enhance specific T cell immune responses against leukemic cells, as well as leukemic progenitor cells, in acute myeloid leukemia. Leukemia.

[86] Wang Y, Rao B, Sun S, Zhu H (2026). Combination therapy of BCL-2 antagonist venetoclax and demethylase inhibitor azacitidine for the treatment of multiple myeloma: a clinical study. Leuk Lymphoma.

[87] Liu S, Zhao Y, Gao Y, Li F, Zhang Y (2024). Targeting metabolism to improve CAR-T cells therapeutic efficacy. Chin Med J.

[88] Choi BD, Yu X, Castano AP, Bouffard AA, Schmidts A, Larson RC (2019). CAR-T cells secreting BiTEs circumvent antigen escape without detectable toxicity. Nat Biotechnol.

[89] Sarangi P (2024). Role of indoleamine 2,3-dioxygenase 1 in immunosuppression of breast cancer. Cancer Pathog Ther.

[90] Feng Q, Sun B, Xue T, Li R, Lin C, Gao Y (2022). Advances in CAR T-cell therapy in bile duct, pancreatic, and gastric cancers. Front Immunol.

[91] Yu B, Xu J, Cui Y (2026). From technological iteration to clinical breakthrough: advances of CAR-T cell therapy in autoimmune diseases. Ann Med.

[92] Wang Y, Cao J, Gu W, Shi M, Lan J, Yan Z (2022). Long-term follow-up of combination of B-cell maturation antigen and CD19 chimeric antigen receptor T cells in multiple myeloma. J Clin Oncol.

[93] Xing R, Wang M, Wang L, Pan M, Wang Y, Zhou H (2024). Clinical updates of B-cell maturation antigen-targeted therapy in multiple myeloma (MM) and relapsed/refractory MM. Int J Mol Med.

[94] Zhang X, Zhu L, Zhang H, Chen S, Xiao Y (2022). CAR-T cell therapy in hematological malignancies: current opportunities and challenges. Front Immunol.

[95] Abbasi S, Totmaj MA, Abbasi M, Hajazimian S, Goleij P, Behroozi J (2023). Chimeric antigen receptor T (CAR-T) cells: novel cell therapy for hematological malignancies. Cancer Med.

[96] Huang J, Huang X, Huang J (2022). CAR-T cell therapy for hematological malignancies: limitations and optimization strategies. Front Immunol.

[97] Ma L, Dou Y, Liu R, Xu T, Yang F, Zheng P (2025). Efficacy and safety of CAR T cell therapy in aggressive B-cell lymphomas involving the gastrointestinal tract. Cancer Rep.

[98] Testa U, Sica S, Pelosi E, Castelli G, Leone G (2024). CAR-T cell therapy in B-cell acute lymphoblastic leukemia. Mediterr J Hematol Infect Dis.

[99] Shalabi H, Qin H, Su A, Yates B, Wolters PL, Steinberg SM (2022). CD19/22 CAR T cells in children and young adults with B-ALL: phase 1 results and development of a novel bicistronic CAR. Blood.

[100] Perez-Amill L, Bataller À, Delgado J, Esteve J, Juan M, Klein-Gonzalez N (2023). Advancing CAR-T therapy for acute myeloid leukemia: recent breakthroughs and strategies for future development. Front Immunol.

[101] Ong MZ, Kimberly SA, Lee WH, Ling M, Lee M, Tan KW (2024). FDA-approved CAR T-cell therapy: a decade of progress and challenges. Curr Pharm Biotechnol.

[102] Tegenge MA, Wang X, Liu J, Zhu H, Fashoyin-Aje LA (2025). FDA experience on CAR T cell pharmacokinetics/pharmacodynamics and model-based assessments. Clin Pharmacol Ther.

[103] Cao LY, Zhao Y, Chen Y, Ma P, Xie JC, Pan XM (2025). CAR-T cell therapy clinical trials: global progress, challenges, and future directions from ClinicalTrials.gov insights. Front Immunol.

[104] Zugasti I, Espinosa-Aroca L, Fidyt K, Mulens-Arias V, Diaz-Beya M, Juan M (2025). CAR-T cell therapy for cancer: current challenges and future directions. Signal Transduct Target Ther.

[105] Khodke P, Hasurkar D, Upadhyay A, Shedge A, Sippy R, Kumbhar BV (2025). B cell antigens: a key to optimizing CAR-T cell therapy. Int Rev Immunol.

[106] Bai Z, Feng B, McClory SE, de Oliveira BC, Diorio C, Gregoire C (2024). Single-cell CAR T atlas reveals type 2 function in 8-year leukaemia remission. Nature.

[107] Mullard A (2025). 2024 FDA approvals. Nat Rev Drug Discov.

[108] Brown CE, Hibbard JC, Alizadeh D, Blanchard MS, Natri HM, Wang D (2024). Locoregional delivery of IL-13Rα2-targeting CAR-T cells in recurrent high-grade glioma: a phase 1 trial. Nat Med.

[109] Bagley SJ, Logun M, Fraietta JA, Wang X, Desai AS, Bagley LJ (2024). Intrathecal bivalent CAR T cells targeting EGFR and IL13Rα2 in recurrent glioblastoma: phase 1 trial interim results. Nat Med.

[110] Shen S, Ruan Z, Jiang B, Qiu W, Zhang F, Shu R (2026). CAR-T cell therapy for pancreatic cancer: translating emerging targets and dual-targeting strategies from solid tumors. Front Immunol.

[111] Norberg SM, Hinrichs CS (2023). Engineered T cell therapy for viral and non-viral epithelial cancers. Cancer Cell.

[112] Azeez SS, Yashooa RK, Smail SW, Salihi A, Ali AS, Mamand S (2025). Advancing CAR-based cell therapies for solid tumours: challenges, therapeutic strategies, and perspectives. Mol Cancer.

[113] Rafii S, Mukherji D, Komaranchath AS, Khalil C, Iqbal F, Abdelwahab SI (2025). Advancing CAR T-cell therapy in solid tumors: current landscape and future directions. Cancers (Basel).

[114] Rosa R, Liu J, Lu C, Abou-el-Enein M, Murad JP, Priceman SJ (2026 Feb 16). Current state of CAR-T cell therapies for solid tumors. Med.

[115] van den Berg J, Läubli H, Khanna N, Jeker LT, Holbro A (2025). Basic concepts and indications of CAR T cells. Hamostaseologie.

[116] Shivani, Agarwal D, Kumar U, Sharma G.

[117] Addani M, Beaman L, Byrd GS, Atala A, Master Z (2025). Toward equitable regenerative medicine: a health equity research framework for emerging regenerative treatments. Regen Med.

[118] Bharadia H, Dabhade A, Shah AC, Patel R, Chorawala MR, Patel A (2025). CAR T-cell immunotherapy as the next horizon in cancer eradication: current landscape, challenges, and future directions. Med Oncol.

[119] Sougiannis MD, Alexander T, Meneses Acosta AN, Ferreira LM (2026). Advancements and challenges in CAR-T cell therapy for cancer treatment. Front Pharmacol.

[120] Mehta R, Jayakumar I (2026). Cytokine release syndrome. J Pediatr Crit Care.

[121] Kim K, Shah R, Ayers A, Ahmed S, Claret L, Strati P (2026). Clinical trial access after CAR T-cell therapy failure in relapsed/refractory large B-cell lymphoma. Br J Haematol.

[122] Zhang Y, Han W (2025). Management of cytokine release syndrome (CRS) following CAR T-cell therapy: a comprehensive review. Clin Cancer Bull.

[123] Gabriel K, Heinzerling L, von Baumgarten L, Subklewe M, Kobold S (2026). The role of cytokines in cytokine release syndrome (CRS) after CAR T cell therapy. Biochim Biophys Acta Mol Cell Res.

[124] Benjamin R, Graham C, Yallop D, Jozwik A, Mirci-Danicar OC, Lucchini G (2020). Genome-edited, donor-derived allogeneic anti-CD19 chimeric antigen receptor T cells in paediatric and adult B-cell acute lymphoblastic leukaemia: results of two phase 1 studies. Lancet.

[125] Ghobadi A, Aldoss I, Maude S, Wayne AS, Bhojwani D, Bajel A (2023). P356:phase 1/2 dose-escalation study of anti-CD7 allogenic CAR-T cell in relapsed or refractory (R/R) T-cell acute lymphoblastic leukemia/lymphoblastic lymphoma (T-ALL/LBL). Hemasphere.

[126] Dimitrov K, Merkle F, Dimitrov M, Merkle S, Hoover A, Bachanova V (2025). Major adverse events with chimeric antigen receptor T-cell therapy: presentation, diagnosis, and resuscitation. Ann Emerg Med.

[127] FDA eliminates risk evaluation and mitigation strategies (REMS) for autologous chimeric antigen receptor (CAR) T cell immunotherapies. https://www.fda.gov/vaccines-blood-biologics/safety-availability-biologics/fda-eliminates-risk-evaluation-and-mitigation-strategies-rems-autologous-chimeric-antigen-receptor.

[128] Mailankody S, Matous JV, Chhabra S, Liedtke M, Sidana S, Oluwole OO (2023). Allogeneic BCMA-targeting CAR T cells in relapsed/refractory multiple myeloma: phase 1 UNIVERSAL trial interim results. Nat Med.

[129] Li YR, Zhu Y, Fang Y, Lyu Z, Yang L (2025). Emerging trends in clinical allogeneic CAR cell therapy. Med.

[130] Jain N, Chevallier P, Liu H, Schiller GJ, Méar JB, DeAngelo DJ (2023). Updated results of the phase I BALLI-01 trial of UCART22 process 2 (P2), an anti-CD22 allogeneic CAR-T cell product manufactured by Cellectis Biologics, in patients with relapsed or refractory (R/R) CD22+ B-cell acute lymphoblastic leukemia (B-ALL). Blood.

[131] Khoury A, Albayeh A, El Halabi L, Mattar B, Reddy PS (2025). Tocilizumab: from bench to bedside—a comprehensive review. Ann Med Clin Oncol.

[132] Cichocki F, Goodridge JP, Bjordahl R, Gaidarova S, Mahmood S, Abujarour R (2021). Off-the-shelf, multiplexed-engineered iPSC-derived NK cells mediate potent multi-antigen targeting of B-cell malignancies with reduced cytotoxicity against healthy B cells. Blood.

[133] Van den Eynde A, Gehrcken L, Verhezen T, Lau HW, Hermans C, Lambrechts H (2024). IL-15-secreting CAR natural killer cells directed toward the pan-cancer target CD70 eliminate both cancer cells and cancer-associated fibroblasts. J Hematol Oncol.

[134] Shah N, Li L, McCarty J, Kaur I, Yvon E, Shaim H (2017). Phase I study of cord blood-derived natural killer cells combined with autologous stem cell transplantation in multiple myeloma. Br J Haematol.

[135] Lee S, Joo Y, Lee EJ, Byeon Y, Kim JH, Pyo KH (2024). Successful expansion and cryopreservation of human natural killer cell line NK-92 for clinical manufacturing. PLoS One.

[136] Marin D, Li Y, Basar R, Rafei H, Daher M, Dou J (2024). Safety, efficacy and determinants of response of allogeneic CD19-specific CAR-NK cells in CD19+ B cell tumors: a phase 1/2 trial. Nat Med.

[137] Liu E, Marin D, Banerjee P, Macapinlac HA, Thompson P, Basar R (2020). Use of CAR-transduced natural killer cells in CD19-positive lymphoid tumors. N Engl J Med.

[138] DiAndreth B, Hamburger AE, Xu H, Kamb A (2022). The Tmod cellular logic gate as a solution for tumor-selective immunotherapy. Clin Immunol.

[139] Punekar SR, Ulrickson M, Specht JM, Wong DJ, Hecht JR, Maus MV (2025). 585 DENALI-1:a seamless phase 1/2 study of A2B395, a logic-gated, allogeneic, Tmod CAR T therapy, in patients with EGFR-expressing solid tumors with human leukocyte antigen-A*02 loss of heterozygosity. J Immunother Cancer.

[140] Liu D, Cao D, Han R (2025). Recent advances in therapeutic gene-editing technologies. Mol Ther.

[141] Strickland SA, Eghtedar A, Farhadfar N, Bajel AR, Ravandi F, Emery R (2025). First-in-human, multicenter study of SENTI-202, a CD33/FLT3 selective off-the-shelf logic-gated CAR NK cell therapy in hematologic malignancies including AML: clinical data. Cancer Res.

[142] Liu Y, Liu G, Wang J, Zheng ZY, Jia L, Rui W (2021). Chimeric STAR receptors using TCR machinery mediate robust responses against solid tumors. Sci Transl Med.

[143] Reddy M, Amrutiya RJ, Win KH (2026). Implementation of CAR-T cell therapy in outpatient settings: a critical review of current literature regarding health outcomes, benefits, challenges, and future directions. Postgrad Med.

[144] Fonkoua LA, Sirpilla O, Sakemura R, Siegler EL, Kenderian SS (2022). CAR T cell therapy and the tumor microenvironment: current challenges and opportunities. Mol Ther Oncolytics.

[145] Lu Y, Zhao F (2025). Strategies to overcome tumour relapse caused by antigen escape after CAR T therapy. Mol Cancer.

[146] Locatelli F, Pagliara D, De Ioris MA, Becilli M, Del Baldo G, Serra A (2025). GD2-targeting CAR T cells in high-risk neuroblastoma: a phase 1/2 trial. Nat Med.

[147] Zhu J, Zhou J, Tang Y, Huang R, Lu C, Qian K (2025). Advancements and challenges in CAR-T cell therapy for solid tumors: a comprehensive review of antigen targets, strategies, and future directions. Cancer Cell Int.

[148] Liao Q, Mao Y, Feng M, Zheng N, Ding X, Zhang X (2025). Advances in CAR-T cell therapy for refractory diseases: challenges, innovations, clinical breakthroughs, and future prospects. Clin Cancer Bull.

[149] Hegde M, Joseph SK, Pashankar F, DeRenzo C, Sanber K, Navai S (2019). Tumor response and endogenous immune reactivity after administration of HER2-directed chimeric antigen receptor T cells in a child with metastatic rhabdomyosarcoma. Nat Commun.

[150] Zhao Z, Sadelain M (2023). CAR T cell design: approaching the elusive AND-gate. Cell Res.

[151] Carcopino C, Erdogan E, Henrich M, Kobold S (2024). Armoring chimeric antigen receptor (CAR) T cells as micropharmacies for cancer therapy. Immuno-Oncol Technol.

[152] Yang DD, Macmorland W, Arnold JN (2025). Current strategies for armoring chimeric antigen receptor T-cells to overcome barriers of the solid tumor microenvironment. Front Immunol.

[153] Qasim W, Zhan H, Samarasinghe S, Adams S, Amrolia P, Stafford S (2017). Molecular remission of infant B-ALL after infusion of universal TALEN gene-edited CAR T cells. Sci Transl Med.

[154] Mehta A, Farooq U, Chen A, McGuirk JP, Ly T, Wong L (2022). Interim phase I clinical data of FT819-101, a study of the first-ever, off-the-shelf, iPSC-derived TCR-less CD19 CAR T-cell therapy for patients with relapsed/refractory B-cell malignancies. Blood.

[155] Ramachandran I, Rothman S, Clausi M, McFadden K, Salantes B, Jih G (2023). Multiple doses of CNTY-101, an iPSC-derived allogeneic CD19 targeting CAR-NK product, are safe and result in tumor microenvironment changes associated with response: a case study. Blood.

[156] Ren T, Wang F, Liu X, Guo J, Xie S (2026). Advances in chimeric antigen receptor-natural killer cell therapy: from mechanisms and preclinical studies to clinical application. Front Oncol.

[157] Hu B, Nastoupil LJ, Holmes H, Hamdan A, Kanate A, Farooq U (2024). A CRISPR-edited allogeneic anti-CD19 CAR-T cell therapy with a PD-1 knockout (CB-010) in patients with relapsed/refractory B cell non-Hodgkin lymphoma (r/r B-NHL): updated phase 1 results from the ANTLER trial. J Clin Oncol.

[158] Su J, Zeng Y, Song Z, Liu Y, Ou K, Wu Y (2025). Genome-edited allogeneic CAR-T cells: the next generation of cancer immunotherapies. J Hematol Oncol.

[159] Mukhametshin SA, Gilyazova EM, Davletshin DR, Ganeeva IA, Zmievskaya EA, Chasov VV (2025). Allogeneic NKG2D CAR-T cell therapy: a promising approach for treating solid tumors. Biomedicines.

[160] Shabaneh TB, Moffett HF, Stull SM, Derezes T, Tait LJ, Park S (2022). Safety switch optimization enhances antibody-mediated elimination of CAR T cells. Front Mol Med.

[161] Olejarz W, Basak G (2023). Emerging therapeutic targets and drug resistance mechanisms in immunotherapy of hematological malignancies. Cancers (Basel).

